# CNS activity of Pokeweed Anti-viral Protein (PAP) in mice infected with Lymphocytic Choriomeningitis Virus (LCMV)

**DOI:** 10.1186/1471-2334-5-9

**Published:** 2005-02-22

**Authors:** Fatih M Uckun, Larisa Rustamova, Alexei O Vassilev, Heather E Tibbles, Alexander S Petkevich

**Affiliations:** 1 Parker Hughes Center for Clinical Immunology, St. Paul, MN 55113, USA; 2 Research Institute for Epidemiology and Microbiology, 220050 MINSK, Belarus

## Abstract

**Background:**

Others and we have previously described the potent *in vivo *and *in vitro *activity of the broad-spectrum antiviral agent PAP (Pokeweed antiviral protein) against a wide range of viruses. The purpose of the present study was to further elucidate the anti-viral spectrum of PAP by examining its effects on the survival of mice challenged with lymphocytic choriomeningitis virus (LCMV).

**Methods:**

We examined the therapeutic effect of PAP in CBA mice inoculated with intracerebral injections of the WE54 strain of LCMV at a 1000 PFU dose level that is lethal to 100% of mice within 7–9 days. Mice were treated either with vehicle or PAP administered intraperitoneally 24 hours prior to, 1 hour prior to and 24 hours, 48 hours 72 hours and 96 hours after virus inoculation.

**Results:**

PAP exhibits significant *in vivo *anti- LCMV activity in mice challenged intracerebrally with an otherwise invariably fatal dose of LCMV. At non-toxic dose levels, PAP significantly prolonged survival in the absence of the majority of disease-associated symptoms. The median survival time of PAP-treated mice was >21 days as opposed to 7 days median survival for the control (p = 0.0069).

**Conclusion:**

Our results presented herein provide unprecedented experimental evidence that PAP exhibits antiviral activity in the CNS of LCMV-infected mice.

## Background

The broad-spectrum anti-viral agent PAP (Pokeweed antiviral protein) [1] is a well-characterized 29-kDa plant-derived ribosome-inactivating protein (RIP) isolated from Phytolacca americana [2]. The anti-viral activity of PAP has been described against a wide range of viruses, including HIV-1, herpes simplex virus, cytomegalovirus, influenza virus and polio virus [2] (For the most recent review, please see: [3]). The activity of PAP is attributed to its ability to inhibit protein synthesis by catalytically cleaving a specific adenine base from the highly conserved alpha-sarcin/ricin loop (SRL) of the large ribosomal RNA [4,5] as well as from viral RNA[3]. The potent anti-HIV activity of PAP at nanomolar ranges taken together with the relative ease of large scale purification has led to the clinical use of PAP [2]. Since 1985, our group has studied the multifunctional efficacy of this potent agent [1,2,4-42].

The purpose of the present study was to further elucidate the anti-viral spectrum of PAP by examining its effects on survival of mice challenged with intracerebral inoculations of lymphocytic choriomeningitis virus (LCMV). LCMV is a rodent-borne arena virus that can result in persistent neuronal infection on mice [43,44]. Alpha-Dystroglycan (alpha-DG) was recently identified as a receptor for LCMV as well as for several other arena viruses including Lassa fever virus [45]. The binding affinity of LCMV to alpha-DG determines viral tropism and the outcome of infection in mice [46]. LCMV has also been associated with both postnatal and intrauterine human disease. Infection in man is acquired after inhalation, ingestion or direct contact with virus found in the urine, feces and saliva of infected mice, hamsters and guinea pigs. Congenital LCMV infection is a significant, often unrecognized cause of chorioretinitis, hydrocephalus, microcephaly or macrocelphaly as well as mental retardation. Acquired LCMV infection, asymptomatic in approximately one third of individuals, is productive of central nervous system manifestations in one half of the remaining cases. Aseptic meningitis or meningoenceophalitis are the predominant syndromes, although transverse myelitis, a Guillain-Barre-type syndrome, as well as transient and permanent acquired hydrocephalus have also been reported [47].

In the present study, we describe the significant efficacy of the highly stable, potent and broad-spectrum anti-viral agent PAP in a murine model of LCMV. Our results presented herein provide unprecedented experimental evidence that PAP exhibits antiviral activity in the CNS of LCMV-infected mice.

## Methods

### Purification of PAP

PAP was purified from spring leaves of the pokeweed plant, *P. americanca*, in four steps [22,38]. Briefly, spring leaves were homogenized in a neutral pH buffer and centrifuged to sediment the remaining cellular fragments. The supernatant was fractionated between 60–90% saturation of ammonium sulfate and the precipitate dialyzed against a low-ionic strength pH 7.5 buffer. The solution was passed through a DEAE cellulose column and the PAP-containing flow-through fraction was then applied to a cation exchange resin S-Sepharose column. The adsorbed PAP was eluted in a linear KCl gradient. The protein peak which eluted at 0.12 KCl was taken as PAP. This fraction was dialyzed extensively against water and lyophilized for storage at -20°C. The procedure resulted in homogeneous PAP, with a purity of >99% as measured by both SDS -12% PAGE and analytical cation- exchange high-performance liquid chromatography. Purified PAP induced concentration-dependent inhibition of HIV-1 replication in normal human peripheral blood mononuclear cells infected with the HIV-1 strain HTLV_IIIB _with an IC_50 _p24 of 14 ± 2 nM.

### Animal infection

Animal infection was performed in an appropriate Animal BioSafety Level-3 Laboratory (ABL-3) at BRIEM (Research Institute for Epidemiology and Microbiology, MINSK, Belarus) with the technician wearing appropriate facility clothing. The culture was thawed in a water bath at 37°C and then diluted in normal saline to achieve the required concentration. In this study, all mice were challenged with 1000 PFU which is 100-times higher than the LD_50_ dose. Each group of animals was placed in a separate cage.

### LCMV model

Three week old CBA mice were intracerebrally infected with 1000 PFU of WE54 strain of LCMV that resulted in lethality of 100% of control (non-treated) animals in 6–8 days after infection. Control animals were given physiologic salt solution as a placebo instead of the compound. In general, for non-treated animals, clinical signs of the disease manifested on the 5^th ^and 7^th ^days by presenting: weight loss, immobility, disheveled hair, convulsions, severe decubitus paralysis and death. All subjective measurement of decreased mobility and scruffy fur were done in a blinded fashion as not to influence the results. The protective properties of the experimental anti-viral drugs were assessed by using the following treatment-preventative regimen: Mice were treated either with vehicle (n = 20) or PAP (n = 10) (0.25 mg/kg) administered intraperitoneally 24 hours prior to, 1 hour prior to, and 24 hours, 48 hours, 72 hours, and 96 hours after virus inoculation. Mice were then observed for 21-days post infection. The protective effect of the experimental anti-viral drugs was evaluated according to the rise of the survival rate and prolongation of mean life of the experimental animals as compared with the control animals.

### Statistical analysis

Statistical significance was determined using the Kaplan Meier Log-Rank test.

## Results

**Figure 1:**
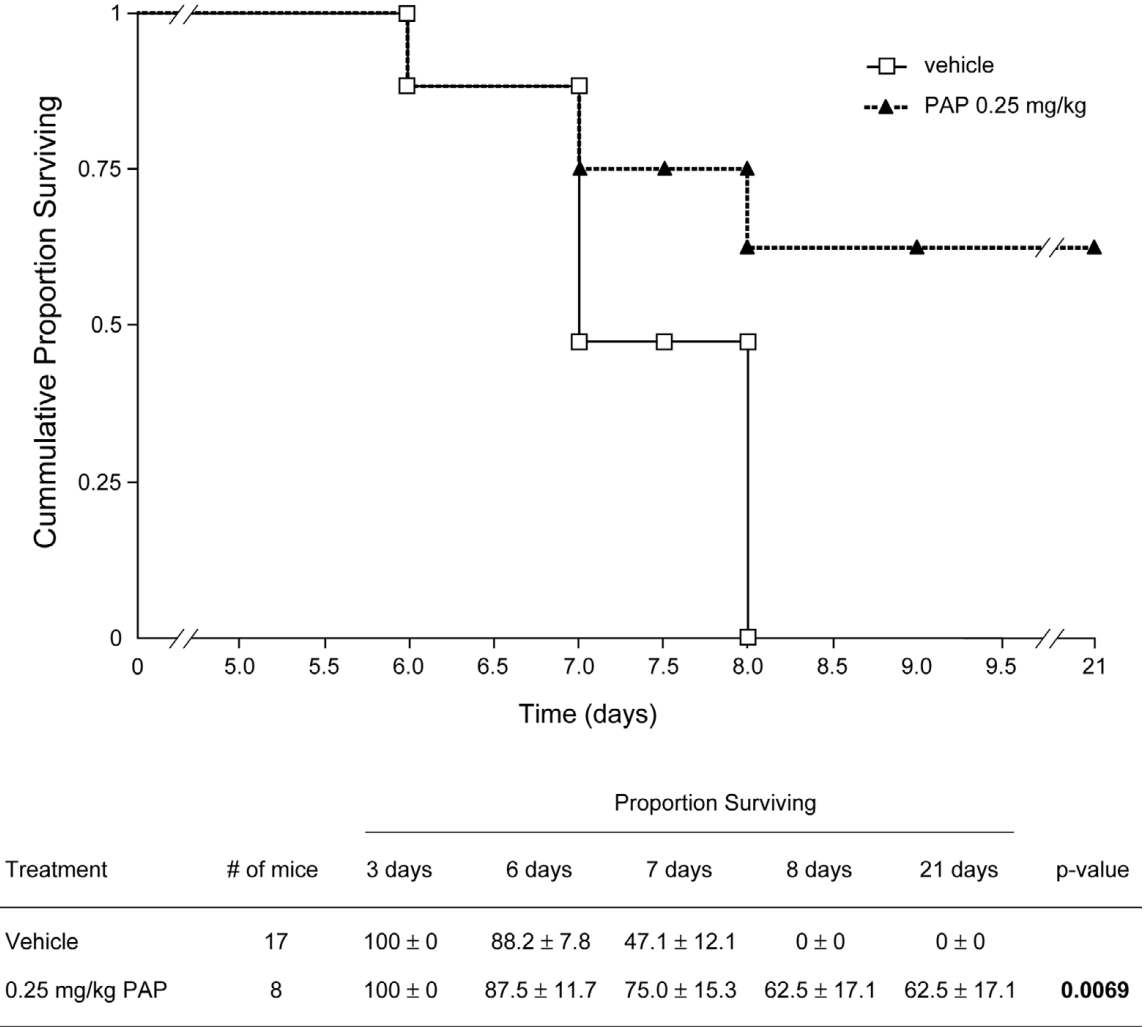
**Protective Activity of PAP in CBA Mice Challenged with LCM Virus**. CBA mice were inoculated with intracerebral injections of the WE54 strain of LCMV at a 1000 PFU dose level that is lethal to 100% of mice within 7–9 days. Mice were treated either with vehicle or PAP administered intraperitoneally 24 hours prior to, 1 hour prior to and 24 hours, 48 hours 72 hours and 96 hours after virus inoculation. Mice were then observed twice daily for 21 days for morbidity and mortality. (*Top*) Shown are representative survival curves detailing the cumulative proportions (%) of mice surviving after virus inoculation. See Table 1 for more detailed information of the treatment outcome. (*Bottom*) Shown is Kaplan-Meir life-table analysis and statistical comparison using the log-rank test.

In order to evaluate the anti-LCMV activity of PAP, mice were inoculated with intracerebral injections of LCMV at a dose level that is lethal to 100% of mice within 6–8 days. Mice were treated either with vehicle or PAP as described in the methods section. Of the 20 control mice, 3 died on day 1 immediately after intracerebral injection due to accidental brain injury and are excluded from the data analysis. All 17 of the remaining control mice developed clinical signs of LCMV infection between day 4 and 6, including weight loss, disheveled hair, decreased mobility and paralysis (Table 1). All control mice developed seizures between day 5 and day7 and died between day 6 and day 8 with a median survival of 7 days (Table 1, Figure 1).

**Table 1:** Anti-LCMV activity of PAP in CBA mice

			**Disease Unset**		
			**Days after inoculation with LCM Virus**		
			
	**Survival (days)**	**Weight loss (grams)**	**Decreased mobility**	**Convulsions**	**Hair disheveled**
	
**Vehicle**					
Mouse #1*	<<1	NA	0	NA	NA
Mouse #2*	<<1	NA	0	NA	NA
Mouse #3*	<<1	NA	0	NA	NA
Mouse #4	6	1.5	4.5	5.5	4.5
Mouse #5	6	2	4.5	5.5	4.5
Mouse #6	7	2	5	6	5
Mouse #7	7	1.5	5	6	5
Mouse #8	7	2.5	5	6	5
Mouse #9	7	4.5	5	6	5
Mouse #10	7	4.5	5	6	5
Mouse #11	7	4.5	5	6	5
Mouse #12	8	2	6	7	6
Mouse #13	8	2.5	6	7	6
Mouse #14	8	1	6	7	6
Mouse #15	8	1.5	6	7	6
Mouse #16	8	2	6	7	6
Mouse #17	8	2	6	7	6
Mouse #18	7	1.5	5	6	5
Mouse #19	8	2	6	7	6
Mouse #20	8	1.5	6	7	6
					
**PAP 0.25 mg/kg**					
Mouse #1	<<1	NA	NA	NA	NA
Mouse #2	<<1	NA	NA	NA	NA
Mouse #3	6	1	4.5	5.5	5
Mouse #4	7	1.5	5	6	5
Mouse #5	8	1.5	6	7	5.5
Mouse #6	21	0	10	NO	NO
Mouse #7	21	0	10	NO	NO
Mouse #8	21	0	10	NO	NO
Mouse #9	21	0	10	NO	NO
Mouse #10	21	0	10	NO	NO

*Mouse died after intracerebral injection; NA = not applicable, NO = not observed

Of the 10 mice treated with the broad-spectrum antiviral agent pokeweed antiviral protein (PAP), 2 died on day 1 immediately after intracerebral injection due to accidental brain injury and are invaluable. Five of the remaining 8 mice (62.5%) treated with PAP remained alive > 21 days post-LCMV inoculation **(median survival >21 days, p = 0.0069) **(Table 1, Figure 1). These mice exhibited no LCMV infection-related weight loss or convulsions and showed no signs of scruffy fur. A significant improvement in mobility was also noted. Thus, PAP exhibited significant *in vivo *anti-LCMV activity in mice challenged intracerebrally with an otherwise invariably fatal dose of LCMV.

## Discussion

In the present study, we describe the significant efficacy of the highly stable, potent and broad-spectrum anti-viral agent PAP in a murine model of LCMV. Our results presented herein provide unprecedented experimental evidence that PAP exhibits antiviral activity in the CNS of LCMV-infected mice. Future studies will examine the therapeutic activity of PAP against LCMV in post-challenge settings. As PAP is well described as an HIV agent, this observation is also relevant as HIV-1 infects the central nervous system (CNS) and it has been feared that the CNS may be a sanctuary site where HIV-1 could hide and continue to replicate despite otherwise effective antiretroviral treatment [48,49]. Antiretroviral therapy of HIV-infected children is associated with a decline in CSF HIV RNA and an improvement in neurological status. The development of genotypic mutations was different in CSF and plasma, suggesting discordant viral evolution. These results suggest that antiretroviral treatment in children should include agents with activity in the CNS [50]. While these results suggest that PAP crosses the blood brain barrier and may therefore be beneficial in other viral infections affecting CNS, they need to be interpreted with due caution for HIV infections involving the CNS since LCMV affects leptomeninges while HIV affects neurons. In future studies, we must also consider the possibility that PAP penetrates the blood brain barrier only when the latter is impaired by the intracerebral administration of the virus. If this is the case, PAP may not be useful in pre-challenge prophylaxis against LCMV-mediated CNS infection. While these results support the notion that the antiviral activity spectrum of PAP covers LCMV as well, an immunomodulatory effect of PAP may also contribute to the observed prophylactic efficacy of PAP against LCMV.

Cases of LCMV infections have been reported in Europe, the Americas, Australia, and Japan. According to the Center for Disease Control, currently there is no specific drug therapy for LCMV. Although the anti-viral agent ribavirin is effective against LCMV in vitro, there is no established evidence to support its use for treatment of LCMV in humans [51].

The anti-viral activity of PAP has been described against numerous pathogenic viruses, which included poliovirus, HIV-1, herpes simplex virus, cytomegalovirus, influenza virus and now, the negative-strand RNA virus Lymphocytic choriomeningitis virus. The ability of PAP to inhibit viral protein synthesis and depurinate viral RNA and DNA [15,16] as well as capped rRNA and mRNAs [52] and its ability to inhibit ribosomal frame shifting and retransposition, make it and ideal candidate for anti-viral strategies.

## Conclusion

Treatment of CBA mice with the broad-spectrum anti-viral agent, PAP significantly improved the probability of survival following the LCMV challenge and decreased overall LCMV-related symptoms. The ability of PAP to exhibit anti-viral activity within the central nervous system is also encouraging within the framework of potential HIV-treatment. We have recently described the rational design and engineering of several recombinant PAP mutants with superior anti-HIV activity [11,17]. In our future studies, we plan to describe the potential anti-LCMV activity of these PAP mutants as well as optimizing the prophylactic and post-exposure treatment regimens. In addition, it will be important to determine if PAP or PAP mutants have activity against other viruses associated with lethal viral hemorrhagic fevers and/or encephalomyelitis, such as the Ebola viruses of the Filoviridae family [53-56].

## List of abbreviations

LCMV: Lymphocytic choriomeningitis virus

PAP: Pokeweed anti-viral protein

HIV and FIV: Human and Feline immunodeficiency virus, respectively

## Competing interests

FMU is listed as an inventor on a number of PAP patents which are owned by PHI. FMU, AV and HET are salaried employees at PHI. PHI is a non-profit public charity.

## Authors' contributions

FMU designed the research project. LR, AP & LT conducted the Lassa experiments. FMU & AV provided PAP and coordinated the Lassa experiments with LR, AP and LT. FMU and HT wrote the manuscript. All authors read and approved manuscript.

## Pre-publication history

The pre-publication history for this paper can be accessed here:


